# Atypical CML with TET2 mutation, associated with NRAS and KRAS: A case report and literature review

**DOI:** 10.1016/j.amsu.2021.102980

**Published:** 2021-10-30

**Authors:** Yousef S. Abuzneid, Hussam I.A. Alzeerelhouseini, Nizar Marzouqa, Yasmine Yaghi, Alaa R. Al-Ihribat, Bilal Alqam, Akram Krama

**Affiliations:** a Al-Quds University Faculty of Medicine, Jerusalem, Palestine; b Istishari Arab Hospital, Ramallah, Palestine; c Palestine Polytechnic University, Hebron, Palestine

**Keywords:** CML, Atypical, CMML, TET2 mutation, Case report

## Abstract

**Background:**

Atypical chronic myeloid leukemia (*BCR-ABL1 negative)* is a rare myeloid neoplasm with poor prognosis and no current standard of treatment. It features both myelodysplastic and myeloproliferative characteristics with little data regarding mutations playing a role in the disease.

**Presentation of case:**

We present a case of a 55-year-old female complaining of fever, cough, general weakness and night sweats. Examinations showed leukocytosis with a left shift, thrombocytopenia, hypercellular bone marrow with marked granulocytic hyperplasia and a negative BCR-ABL. After ruling out myelodysplastic and other myeloproliferative diseases the patient was finally diagnosed as aCML according to the WHO criteria with mutations in the TET2 gene, the NRAS gene and in the KRAS gene. The patient was started on Hydroxyurea for a duration of 9 months with an excellent initial response leading to normalization of her platelets and WBCs. However, in the last month she stopped responding to therapy and her state of health started declining once again.

**Conclusion:**

Atypical chronic myeloid leukemia (*BCR-ABL1 negative* with presence of TET2 gene mutation) is an unusual finding in myeloid neoplasms, have unknown prognosis and no current standard of treatment. It features both myelodysplastic and myeloproliferative characteristics with little data regarding mutations playing a role in the disease.

## Highlights

• Atypical CML is challenging to diagnose and can be misdiagnosed with CMML.
• Careful history taking, meticulous physical examination, and investigations are necessary.
• A treatment is not yet stipulated; however, researchers are working on it.

## 1 Introduction

Atypical chronic myeloid leukemia (aCML) is a rare neoplasm of hematopoietic stem cells that falls within the category of myelodysplastic/myeloproliferative syndromes diseases (MDS/MPN) due to its overlapping myelodysplastic and myeloproliferative features [1].

According to the WHO diagnostic criteria, aCML shows negative BCR-ABL1 fusion gene on cytogenic and molecular studies, dysgranulopoiesis, persistent leukocytosis with immature granulocytes accounting for >10% of all leukocytes, <20% blasts in the peripheral blood and bone marrow and minimal/absent monocytosis. It is a disorder of the elderly, mainly occurring in the 7th or 8th decade of life, with no apparent sex predominance and an incidence of 1–2 cases for every 100 cases of Philadelphia-positive CML [2].

Overall, aCML is highly associated with poor prognosis with a median survival of less than two years and a 20–40% chance of evolving to acute myeloid leukemia [2]. Although uncommon, aCML is an aggressive disease with no standard care of treatment. Allogeneic hematopoietic stem cell transplantation (HSCT) offers the only potentially curative option [3].

The work has been reported in line with the CARE criteria [17].

## 2 Case presentation

A 53-year-old female patient was in her usual state of health until September 2019 when she started to complain from recurrent episodes of fever associated with cough, night sweats and general weakness. She sought medical advice and laboratory tests were done which showed leukocytosis with a WBC count of 37.85 x 10^3^/μL (normal range: 4.1–10.8 x 10^3^/μL), an increase in neutrophils 85.1% (normal range 54–62%) and decrease in lymphocytes 8.5% (normal range: 25–33%) and thrombocytopenia 127 x 10^3^/μL (normal range 150-350 x 10^3^/μL). Her blood film showed neutropenia with a left shift with normal RBCs and platelets (PLT). Immunochemistry for ANA-HEP-2 was negative. An abdominal ultrasound scan was also preformed but with unremarkable results. Hence, she was referred to a specialist for evaluation. A hematological blood film report on 25th September revealed normocytic, normochromic RBCs with slight rouleax; leukocytosis (segmented neutrophils, eosinophils, basophils and monocytes were seen) with the neutrophils in band and myelocytes present making a diagnosis of CML highly suspected. Multiple myeloma had to be excluded so a bone marrow biopsy was performed in November 2019 which showed no evidence of increased plasma cells by CD138 stain. Thus, a real-time PCR was done for major and minor BCR-ABL fusion transcripts t(9; 22)(q34; q11) but it did not detect any abnormalities.

**Fig. 1:**
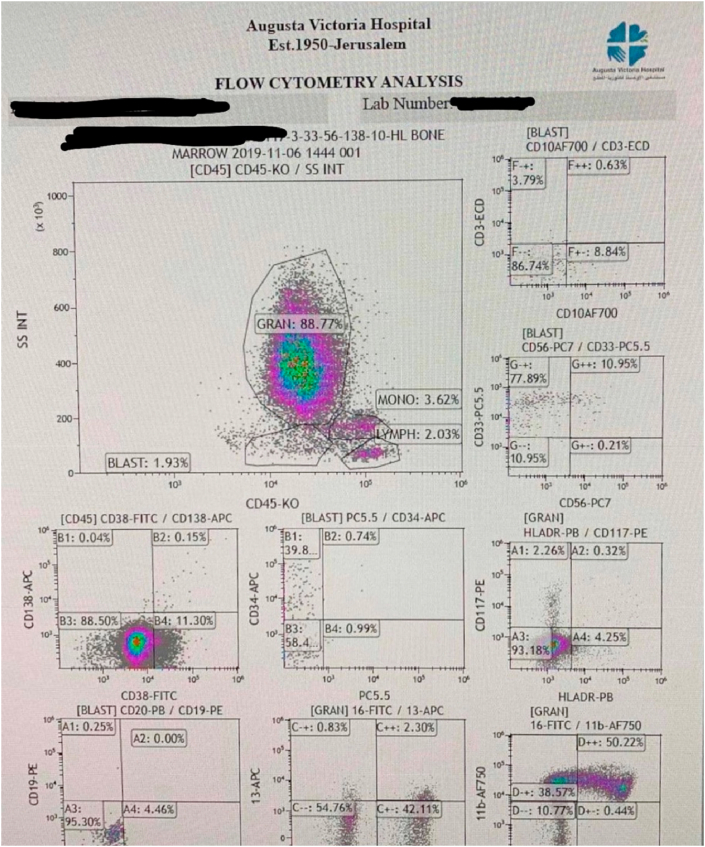
Patient's flow cytometry showing that the blasts were 2%, the granulocytes were 89%, the lymphocytes were 2% and the monocytes were 3.6%.

A flow cytometry analysis was then performed which showed: 2% blasts, 89% granulocytes, 2% lymphocytes and 3.6% monocytes 3.6% showing no evidence of bone marrow involvement by myeloma cells (Fig. 1).

**Fig. 2:**
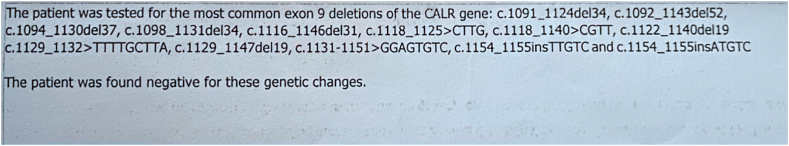
Results from the CALR Exon 9 mutation analysis.

On 5^th^ February 2020, and without any clue of a diagnosis for the patient, she was asked to do a CALR Exon 9 mutation analysis, a PCR test followed by Sanger sequencing, to rule out any myeloproliferative neoplasms but the results were negative (Fig. 2).

In the same month, she was asked to do a MPL1 W515L/K testing by the same method of PCR and Sanger sequencing that turned out to be negative as well.

Subsequently, she was asked to do a BCR-ABL major fusion gene (p210) quantification test, in which total RNA extraction form patient's WBC followed by qPCR on Rotor-Gene real time PCR instrument from QIAGEN was done. However, it was not detected.

A serum protein electrophoresis was subsequently done on 10th February, showing a decrease in albumin (48.6% when the normal range is 54–66%), slight increase in alpha 1 protein (2.9% when the normal range is 1.4–2.8%) and increase in gamma protein (26.2% when the normal range is 10.6–19.2%).

She was referred to our hospital on 26th February since there was no clue for her diagnosis and all the laboratory tests were negative. Laboratory studies were redone as an initial step which exhibited leukocytosis and thrombocytopenia as the previous ones (WBC were 90.12 when the normal range is 4-11 x 10^3^/μL, neutrophils were 74.04 when the normal range is 2–8.7 x 10^3^/μL, lymphocytes percentage was decrease, neutrophils percentage was increased and PLTs were 90 when the normal range is 140–440). Also, our studies showed basophilia with 0.2% (normal range: 0.0–0.1%) and a basophil count of 0.15 (when the normal range is 0.0–0.1 x 10^3^/μL). This was followed by a BCR-ABL t(9; 22) translocation quantitative test by RT-PCR that was negative for major and minor criteria (same results as the tests performed previously).

As a first treatment, she was given Hydroxyurea (20 mg 1 x 1) and further laboratory tests were asked to be done.

We did a bone marrow aspirate that was hemodiluted, but we could not assess a stainable iron due to lack of particles. In addition, a bone marrow biopsy was performed that exhibited a hypercellular bone marrow with marked granulocytic hyperplasia with no morphologic evidence of acute leukemia.

The reticulin stain, done later on 10th March, was grade 1 fibrosis, the cellularity was 95%, granulocytic precursors were markedly increased, the megakaryocytes were decreased, and the lymphocytes and plasma cells were normal).

Immunochemistry tests for MPO, CD34 and CD99 were done, but they were negative.

A day later, a bone marrow biopsy was executed showing that the major BCR-ABL was not detected.

With all this information, it was suspected that the patient was a case of atypical chronic myelogenous leukemia (aCML) since the patient was negative for BCR-ABL t(9; 22) genetic mutation, also known as Philadelphia chromosome, had persistent leukocytosis with absent monocytosis, exhibited a hypercellular bone marrow with granulocytic hyperplasia, had 2% blasts and presented with increased basophil count.

In June, an exome genetic study showed that the patient had mutations in the TET2 gene (a mutation in exon 3, in the nucleotide NM_001127208.2:c.1595delT, in the amino acid p.L532*), the NRAS gene (a mutation in exon 2, in the nucleotide NM_002524.5:c.35G > A, in the amino acid p.G12D) and in the KRAS gene (a mutation in exon 2, in the nucleotide NM_033360.4:c.35G > T, in the amino acid p.G12V).

**Fig. 3:**
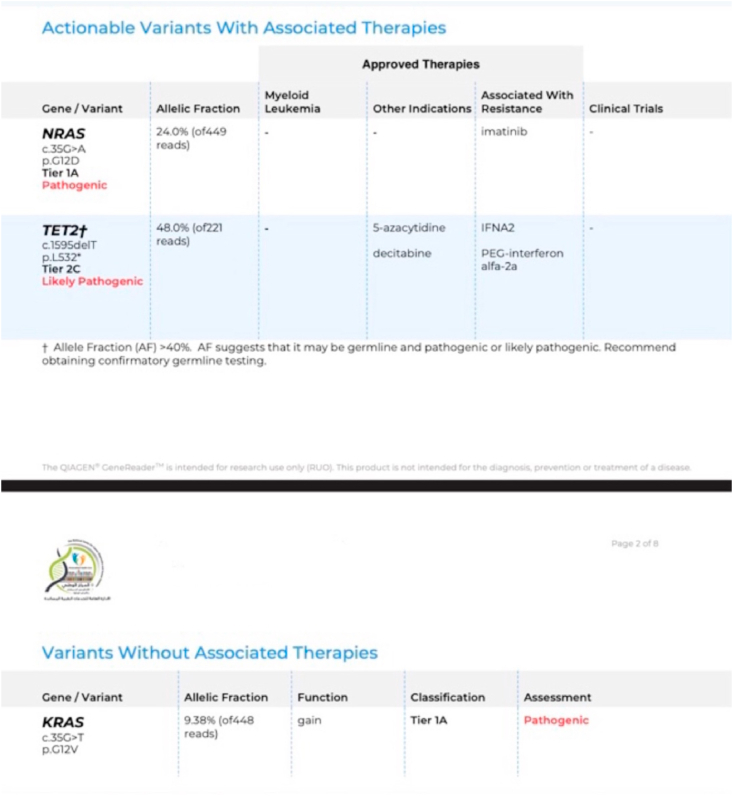
Results from the patient's exome genetic study.

According to our results, the mutations in the KRAS and NRAS genes were classified as tier 1A, which means that both mutations are pathogenic; whereas the TET2 gene mutation was classified as tier 2C which means that is likely pathogenic (Fig. 3).

Further follow-ups were scheduled for the patient. In October 2020, an abdominal ultrasound was preformed which excluded organomegaly, paraaortic lymphadenopathy and free fluid in the upper abdomen.

During the follow up, her CBC displayed surprising results with normal WBC and the platelets count (WBC were 9.24 x 10^3^/μL and the PLT were 229 x 10^3^/μL). Furthermore, the basophils percentage dropped to 0.16%.

Apparently, the patient was reacting well to Hydroxyurea despite the mechanism not being fully understood. Unfortunately, a CBC on November 2020 showed a deterioration in her health with an increase in WBC and a decrease in platelets (WBC were 21.59 x 10^3^/μL and the platelets were 176 x 10^3^/μL) and with an elevation in the basophil count (0.22 when the normal range is 0.0–0.2 x 10^3^/μL). Furthermore, an abdominal ultrasound scan was performed with had unremarkable results.

Recently we also added azacytidine to the management of our patient as we saw in a case report from 2019 with some similarities [18], but still we did not notice any improvement on the symptomatology.

## 3 Discussion

Chronic myeloid leukemia (CML) is a disease that for many years has been defined as BCR-ABL1 positive disease. Included in this disease, there is a condition defined as a poor prognosis, clinically heterogeneous entity termed ‘BCR-ABL1 negative CML’ constituting about 5% of CML cases. Other conditions, but a minority, could be classified as systemic mastocytosis with associated hematological neoplasm (SM-AHN), myeloid/lymphoid neoplasms associated with eosinophilia and rearrangement of PDGFRA, PDGFRB, FGFR1 or with PCM1-JAK2 (MLN-eo), or chronic eosinophilic leukemia not otherwise specified (CEL-NOS) [4].

Atypical chronic myeloid leukemia (aCML), which is BCR-ABL1 negative, is a rare hematological malignancy that falls within the overlap category of myelodys-plastic/myeloproliferative neoplasms (MDS/MPN) in the WHO classification of myeloid neoplasms. It was initially described as an ‘atypical’ form of CML, which is BCR-ABL1 positive, leading to confusing; however, aCML is now known to be an entirely separate entity from CML [7,10].

The diagnostic criteria for aCML have significantly changed over and it is now known to have both a myeloproliferative component with significant leukocytosis and often splenomegaly and hepatomegaly, as well as myelodysplastic components, most strikingly in the granulocytic lineage [5].

Features of aCML include splenomegaly, myeloid dysplasia, leukocytosis (WBC >13 k/ml) with immature granulocytes accounting for >10% of all leukocytes, <20% blasts in the peripheral blood and bone marrow, and lack of sustained monocytosis [11].

The molecular features of aCML include an increased frequency of gene fusions or an aneuploid karyotype. One or two chromosomal aberrations (like trisomy 8 or 9, del (20q), −7/7q or isochromosomes 17q) are found in up to 50% of patients but are non-specific and similar to cytogenetic findings detectable also in MDS [6].

There is no specific data regarding the immunophenotype of aCML. However, immunophenotyping of peripheral blood and bone marrow for CD14, CD68R, and/or CD163 may facilitate monocyte quantification in cases where both aCML and CMML are in the differential diagnosis. Flow cytometric and immunohistochemical evaluation of monocytes may be impacted by alterations of antigen expression, being helpful for the diagnosis of this condition [16].

There are other immunohistochemical findings in bone marrow core biopsies that can be used as an alternative to counting monocytes when attempting to distinguish aCML from CMML. For example, immunohistochemical staining for CD123 can be used to identify plasmacytoid dendritic cell nodules on bone marrow core biopsies, which are a specific (although somewhat insensitive) finding that favors a diagnosis of CMML [7,15].

There is currently no cure or standard of care for the management of aCML. Its heterogeneous clinical and genetic features, high rate of transformation to AML with historically poor survival, and absence of robust randomized clinical trial data to support treatment recommendations are a challenge in determining the appropriate management of this disease [8].

The Ten-Eleven-Translocation 2 (TET2) gene encodes a member of TET family enzymes that alters the epigenetic status of DNA by oxidizing 5-methylcytosine to 5 hydroxymethylcytosine (5hmC). Somatic loss-of-function mutations of TET2 are frequently observed in patients with diverse myeloid malignancies, including myelodysplastic syndromes, myeloproliferative neoplasms, and chronic myelomonocytic leukemia [12,13].

Regarding aCML, mutational frequencies such as ASXL1 28%, TET2 16%, NRAS 16%, SETBP1 12%, RUNX1 12%, ETNK1 8% and PTPN11 4% are seen in many papers [9,13].

The exact prevalence and impact of TET2 in aCML remains to be elucidated. *Patnaik* et al. [14] stipulate in his paper that 16% of the patients had TET2 mutations. The presence of these mutations independently and adversely impacted in patient's survival. Increasing age, progressive anemia and the presence of TET2 mutations adversely impacted survival, providing an effective risk stratification system for affected patients.

As we could see in the case presentation, aCML was a diagnosis of exclusion; we discarded other possible clinical abnormalities with laboratory tests, bone marrow biopsies and genetic tests such as acute leukemia, multiple myeloma, myelodysplastic diseases and typical CML.

Through an exome genetic test to discard any mutation, we could explain a possible diagnosis of aCML since we found that our patient has a mutation in the TET2 gene and also mutations in the NRAS and KRAS that were likely pathogenic for this condition, making the diagnosis clearer.

Until now, we do not understand the mechanisms or have an explanation for the mild improvement of our patient with Hydroxyurea but a possible explanation is that it affected the immune system of our patient causing mild improvement for a short period of time.

## 4 Conclusion

aCML remains a diagnosis of exclusion, in which the workup, as we've seen in this case, is extensive. However, it should be kept in mind when facing cases of suspected chronic leukemias and MDS/MPS, raising suspicion if a BCR-ABL1 test turned out negative. In our case, the patient, positive for TET2, KRAS, NRAS mutations, only briefly benefited on Prednitab. For this reason, we think that more research is needed to study the effectiveness of possible aCML treatment modalities.

## Ethical approval

The study is exempt from ethical approval in our institution.

## Sources of funding

No funding or grant support.

## Author contribution

Study concept or design: Akram Krama.

Writing the manuscript: Yousef S. Abuzneid, Hussam I. A. Alzeerelhouseini, Nizar Marzouqa, Yasmine Yaghi, Alaa R. Al-Ihribat, Bilal Alqam.

Review & editing the manuscript: Yousef S. Abuzneid, Hussam I. A. Alzeerelhouseini.

## Consent

Written informed consent was obtained from the patient for publication of this case report and accompanying images. A copy of the written consent is available for review by the Editor-in-Chief of this journal on request.

## Registration of research studies

Not applicable.

## Guarantor

Dr. Yousef S. Abuzneid.

## Provenance and peer review

Not commissioned, externally peer-reviewed.

## Declaration of competing interest

There is no conflict of interest.

## References

[1] Wang S.A., Hasserjian R.P., Fox P.S., Rogers H.J., Geyer J.T., Chabot-Richards D., Weinzierl E., Hatem J., Jaso J., Kanagal-Shamanna R., Stingo F.C., Patel K.P., Mehrotra M., Bueso-Ramos C., Young K.H., Dinardo C.D., Verstovsek S., Tiu R.V., Bagg A., Hsi E.D., Arber D.A., Foucar K., Luthra R., Orazi A. (2014 Apr 24). Atypical chronic myeloid leukemia is clinically distinct from unclassifiable myelodysplastic/myeloproliferative neoplasms. Blood.

[2] Belkhair J., Raissi A., Elyahyaoui H., Ameur M.A., Chakour M. (2019 May 25). Atypical chronic myeloid leukemia BCR-ABL 1 negative: a case report and literature review. Leuk Res Rep.

[3] Onida F., de Wreede L.C., van Biezen A., Eikema D.J., Byrne J.L., Iori A.P., Schots R., Jungova A., Schetelig J., Finke J., Veelken H., Johansson J.E., Craddock C., Stelljes M., Theobald M., Holler E., Schanz U., Schaap N., Bittenbring J., Olavarria E., Chalandon Y., Kröger N. (2017 Jun). Allogeneic stem cell transplantation in patients with atypical chronic myeloid leukaemia: a retrospective study from the Chronic Malignancies Working Party of the European Society for Blood and Marrow Transplantation. Br. J. Haematol..

[4] Cross N.C.P. (2020 Sep). Update on CML-like disorders. Clin. Lymphoma, Myeloma & Leukemia.

[5] Sadigh S., Hasserjian R.P., Hobbs G. (2020 Mar). Distinguishing atypical chronic myeloid leukemia from other Philadelphia-negative chronic myeloproliferative neoplasms. Curr. Opin. Hematol..

[6] Crisà E., Nicolosi M., Ferri V., Favini C., Gaidano G., Patriarca A. (2020 Sep 18). Atypical chronic myeloid leukemia: where are we now?. Int. J. Mol. Sci..

[7] Levinson Katherine, Bagg Adam (2018). Atypical chronic myeloid leukemia. BCR/ABL1 Negative.

[8] Schwartz L.C., Mascarenhas J. (2019 Jan). Current and evolving understanding of atypical chronic myeloid leukemia. Blood Rev..

[9] Gao T., Yu C., Xia S., Liang T., Gu X., Liu Z. (2020 Jul 28). A rare atypical chronic myeloid leukemia BCR-ABL1 negative with concomitant *JAK2* V617F and *SETBP1* mutations: a case report and literature review. Ther. Adv. Hematol..

[10] Onida F., Ball G., Kantarjian H.M., Smith T.L., Glassman A., Albitar M., Scappini B., Rios M.B., Keating M.J., Beran M. (2002 Oct 15). Characteristics and outcome of patients with Philadelphia chromosome negative, bcr/abl negative chronic myelogenous leukemia. Cancer.

[11] Freedman J.L., Desai A.V., Bailey L.C., Aplenc R., Burnworth B., Zehentner B.K., Teachey D.T., Wertheim G. (2016 Jan). Atypical chronic myeloid leukemia in two pediatric patients. Pediatr. Blood Cancer.

[12] Ko M., Bandukwala H.S., An J., Lamperti E.D., Thompson E.C., Hastie R., Tsangaratou A., Rajewsky K., Koralov S.B., Rao A. (2011 Aug 30). Ten-Eleven-Translocation 2 (TET2) negatively regulates homeostasis and differentiation of hematopoietic stem cells in mice. Proc. Natl. Acad. Sci. U. S. A..

[13] Solary E., Bernard O.A., Tefferi A., Fuks F., Vainchenker W. (2014 Mar). The Ten-Eleven Translocation-2 (TET2) gene in hematopoiesis and hematopoietic diseases. Leukemia.

[14] Patnaik M.M., Barraco D., Lasho T.L., Finke C.M., Reichard K., Hoversten K.P., Ketterling R.P., Gangat N., Tefferi A. (2017 Jun). Targeted next generation sequencing and identification of risk factors in World Health Organization defined atypical chronic myeloid leukemia. Am. J. Hematol..

[15] Pronier E., Delhommeau F. (2012 Mar). Role of TET2 mutations in myeloproliferative neoplasms. Curr. Hematol. Malig. Rep..

[16] Meggendorfer M., Haferlach T., Alpermann T., Jeromin S., Haferlach C., Kern W., Schnittger S. (2014 Dec). Specific molecular mutation patterns delineate chronic neutrophilic leukemia, atypical chronic myeloid leukemia, and chronic myelomonocytic leukemia. Haematologica.

[17] Riley D.S., Barber M.S., Kienle G.S., AronsonJK (2017 Sep). CARE guidelines for case reports: explanation and elaboration document. JClinEpi.

[18] Marumo A., Mizuki T., Tanosaki S. (2019 Oct-Dec). Atypical chronic myeloid leukemia achieving good response with azacitidine. Indian J. Cancer.

